# *catena*-Poly[[(benzoato-κ^2^ *O*,*O*′)(2,2′-bipyridine-κ^2^ *N*,*N*′)lead(II)]-μ_3_-nitrato-κ^4^ *O*:*O*,*O*′:*O*′′]

**DOI:** 10.1107/S160053681004907X

**Published:** 2010-11-27

**Authors:** Juan Yang, Jun Dai, Xiaohan Wang

**Affiliations:** a Department of Physical Chemistry, Henan Polytechnic University, Jiaozuo 454003, People’s Republic of China; b Institute of Safety Science and Engineering, Henan Polytechnic University, Jiaozuo 454003, People’s Republic of China

## Abstract

In the title coordination polymer, [Pb(C_7_H_5_O_2_)(NO_3_)(C_10_H_8_N_2_)]_*n*_, the Pb^II^ ion is eight-coordinated by two N atoms from one 2,2′-bipyridine ligand, two O atoms from one benzoate anion and four O atoms from three nitrate groups (one chelating, two bridging) in a distorted dodecahedral geometry. Adjacent Pb^II^ ions are linked by bridging nitrate O atoms through the central Pb_2_O_2_ and Pb_2_O_4_N_2_ cores, resulting in an infinite chain structure along the *b* axis. The crystal structure is stabilized by π–π stacking inter­actions between 2,2′-bipyridine and benzoate ligands belonging to neighboring chains, with shortest centroid–centroid distances of 3.685 (8) and 3.564 (8) Å.

## Related literature

For applications of complexes containing Pb(II), see: Fan & Zhu (2006 ▶); Hamilton *et al.* (2004 ▶); Alvarado *et al.* (2005 ▶). For the use of aromatic carboxyl­ate and 2,2′-bipyridine-like ligands in the preparation of metal-organic complexes, see: Wang *et al.* (2006 ▶); Masaoka *et al.* (2001 ▶); Hagrman & Zubieta (2000 ▶); Li *et al.* (2002 ▶).
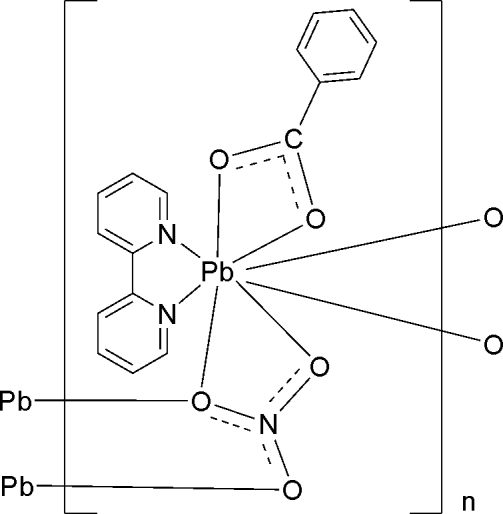


## Experimental

### 

#### Crystal data

[Pb(C_7_H_5_O_2_)(NO_3_)(C_10_H_8_N_2_)]
*M* *_r_* = 546.49
Triclinic, 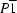

*a* = 6.5389 (11) Å
*b* = 8.5052 (14) Å
*c* = 15.548 (3) Å
α = 84.566 (3)°
β = 86.593 (3)°
γ = 83.729 (2)°
*V* = 854.6 (3) Å^3^
*Z* = 2
Mo *K*α radiation
μ = 9.91 mm^−1^
*T* = 296 K
0.23 × 0.21 × 0.15 mm

#### Data collection

Bruker APEXII CCD area-detector diffractometer
Absorption correction: multi-scan (*SADABS*; Bruker, 2007 ▶) *T*_min_ = 0.118, *T*_max_ = 0.226
4385 measured reflections
2981 independent reflections
2769 reflections with *I* > 2σ(*I*)
*R*_int_ = 0.030

#### Refinement

*R*[*F* ^2^ > 2σ(*F* ^2^)] = 0.037
*wR*(*F* ^2^) = 0.099
*S* = 1.02
2981 reflections
235 parameters
H-atom parameters constrained
Δρ_max_ = 2.70 e Å^−3^
Δρ_min_ = −2.79 e Å^−3^

### 

Data collection: *APEX2* (Bruker, 2007 ▶); cell refinement: *SAINT* (Bruker, 2007 ▶); data reduction: *SAINT*; program(s) used to solve structure: *SHELXS97* (Sheldrick, 2008 ▶); program(s) used to refine structure: *SHELXL97* (Sheldrick, 2008 ▶); molecular graphics: *SHELXTL* (Sheldrick, 2008 ▶); software used to prepare material for publication: *SHELXTL*.

## Supplementary Material

Crystal structure: contains datablocks global, I. DOI: 10.1107/S160053681004907X/gk2325sup1.cif

Structure factors: contains datablocks I. DOI: 10.1107/S160053681004907X/gk2325Isup2.hkl

Additional supplementary materials:  crystallographic information; 3D view; checkCIF report

## Figures and Tables

**Table 1:** Selected bond lengths (Å)

Pb1—O1	2.432 (5)
Pb1—N2	2.441 (6)
Pb1—N1	2.471 (5)
Pb1—O2	2.619 (6)
Pb1—O4	2.871 (6)
Pb1—O3	2.928 (6)
Pb1—O3^i^	2.893 (6)
Pb1—O5^ii^	2.887 (7)

Symmetry codes: (i) 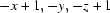; (ii) 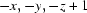.

**Fig. 1.**
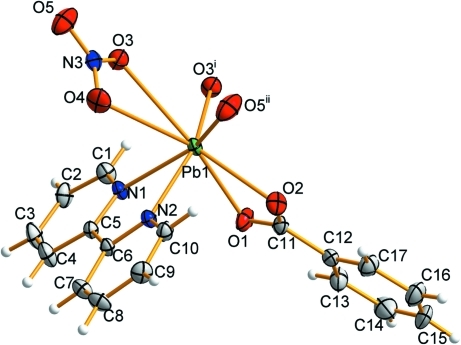
The coordination environment around PbII in the title compound with the atom-labeling scheme. Displacement ellipsoids for non-hydrogen atoms are drawn at the 30% probability level. Symmetry codes: (i) 1-x, -y, 1-z; (ii) -x, -y, 1-z.

**Fig. 2.**
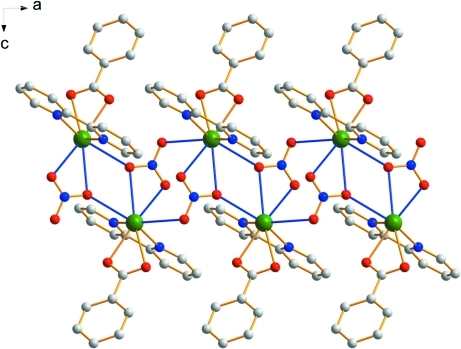
The chain of the title polymer viewed down the b axis.; H atoms are omitted for clarity.

## supplementary crystallographic information

### Comment

Complexes containing Pb(II) ion have recently attracted considerable interest not only because of the variety of their architectures, but also because of their potential applications, especially in environmental protection and in systems with different biological properties (Fan & Zhu, 2006; Hamilton *et al.*, 2004; Alvarado *et al.*, 2005). As an important family of multidentate O-donor ligands, aromatic carboxylate ligands have been extensively employed in the preparation of metal-organic complexes because of their potential properties and intriguing structural topologies (Wang *et al.*, 2006; Masaoka *et al.*, 2001). To our knowledge, carboxylate coordinates metal in various ways, for example, in the mode of monodentate, bidentate chelating, bidentate bridging or chelating-bridging. It is well known that the introduction of chelate ligands such as 2,2'-bipyridine are capable of passivating metal sites via the N donors of the organic groups and may induce new structural evolution (Hagrman *et al.*, 2000; Li *et al.*, 2002). Herein, we report the structure of the title polymer.

The asymmetric unit of the title compound, [Pb(C_10_H_8_N_2_)(C_7_H_5_O_2_)(NO_3_)], contains a Pb^II^ cation, one 2,2'-bipyridine ligand, one benzoate and one nitrate ligand, as illustrated in in Fig.1. The Pb^II^ atom is eight-coordinated by two N atoms from one 2,2'-bipyridine ligand, two O atoms from one benzoate anion and four O atoms from three chelating-bridging nitrate ligands in a distorted dodecahedron geometry. The O3 and O5 atoms of bridging nitrato ligands link the adjacent Pb^II^ ions through the central Pb_2_O_2_ and Pb_2_O_4_N_2_ cores, resulting in an infinite chain structure along the *b* axis (Fig.2). The excellent coordinating ability and large conjugated systems of 2,2'-bipyridine and benzoato ligands allow to form π..π interactions. The chains are extended into the framework through π..π stacking interactions between the ligands belonging to the neighboring chains, with the shortest centroid-centroid distance of 3.685 (8) and 3.564 (8)Å.

### Experimental

A mixture of Pb(NO_3_)_2_ (0.09 g, 0.27 mmol), benzoic acid (0.102 g, 0.84 mmol), 2,2'-bipyridine (0.065 g, 0.41 mmol) and distilled water (10 ml) was sealed in a 25 ml Teflon-lined stainless autoclave. The mixture was heated at 403 K for 6 days to give the colorless crystals suitable for X-ray diffraction analysis.

### Refinement

All H atoms bounded to C atoms were placed in calculated positions and treated in a riding-model approximation, with C—H = 0.93 Å and *U*_iso_(H) = 1.2U_eq_(C).

### Crystal data

**Table:**

[Pb(C_7_H_5_O_2_)(NO_3_)(C_10_H_8_N_2_)]	*Z* = 2
*M**_r_* = 546.49	*F*(000) = 516
Triclinic, *P*1	*D*_x_ = 2.124 Mg m^−^^3^
Hall symbol: -P 1	Mo *K*α radiation, λ = 0.71073 Å
*a* = 6.5389 (11) Å	Cell parameters from 3772 reflections
*b* = 8.5052 (14) Å	θ = 2.6–28.2°
*c* = 15.548 (3) Å	µ = 9.90 mm^−^^1^
α = 84.566 (3)°	*T* = 296 K
β = 86.593 (3)°	Column, colorless
γ = 83.729 (2)°	0.23 × 0.21 × 0.15 mm
*V* = 854.6 (3) Å^3^	

### Data collection

**Table:**

Bruker APEXII CCD area-detector diffractometer	2981 independent reflections
Radiation source: fine-focus sealed tube	2769 reflections with *I* > 2σ(*I*)
graphite	*R*_int_ = 0.030
φ and ω scans	θ_max_ = 25.0°, θ_min_ = 2.7°
Absorption correction: multi-scan (*SADABS*; Bruker, 2007)	*h* = −7→7
*T*_min_ = 0.118, *T*_max_ = 0.226	*k* = −9→10
4385 measured reflections	*l* = −18→15

### Refinement

**Table:**

Refinement on *F*^2^	Primary atom site location: structure-invariant direct methods
Least-squares matrix: full	Secondary atom site location: difference Fourier map
*R*[*F*^2^ > 2σ(*F*^2^)] = 0.037	Hydrogen site location: inferred from neighbouring sites
*wR*(*F*^2^) = 0.099	H-atom parameters constrained
*S* = 1.02	*w* = 1/[σ^2^(*F*_o_^2^) + (0.0675*P*)^2^] where *P* = (*F*_o_^2^ + 2*F*_c_^2^)/3
2981 reflections	(Δ/σ)_max_ = 0.001
235 parameters	Δρ_max_ = 2.70 e Å^−^^3^
0 restraints	Δρ_min_ = −2.79 e Å^−^^3^

### Special details

**Table:**

Experimental. Refinement Refinement of F^2^ against ALL reflections. The weighted R-factor wR and goodness of fit S are based on F^2^, conventional R-factors R are based on F, with F set to zero for negative F^2^. The threshold expression of F^2^ > 2sigma(F^2^) is used only for calculating R-factors(gt) etc. and is not relevant to the choice of reflections for refinement. R-factors based on F^2^ are statistically about twice as large as those based on F, and R- factors based on ALL data will be even larger. Geometry All e.s.d.'s (except the e.s.d. in the dihedral angle between two l.s. planes) are estimated using the full covariance matrix. The cell e.s.d.'s are taken into account individually in the estimation of e.s.d.'s in distances, angles and torsion angles; correlations between e.s.d.'s in cell parameters are only used when they are defined by crystal symmetry. An approximate (isotropic) treatment of cell e.s.d.'s is used for estimating e.s.d.'s involving l.s. planes.

### Fractional atomic coordinates and isotropic or equivalent isotropic displacement parameters (Å^2^)

**Table:**

	*x*	*y*	*z*	*U*_iso_*/*U*_eq_	
Pb1	0.31380 (3)	0.02444 (2)	0.362929 (15)	0.02612 (13)	
O3	0.3180 (9)	0.0845 (7)	0.5451 (4)	0.0433 (14)	
O2	0.2641 (8)	−0.0974 (7)	0.2180 (4)	0.0409 (13)	
O4	0.0370 (9)	0.1789 (8)	0.4866 (4)	0.0546 (16)	
O1	0.5402 (9)	0.0249 (7)	0.2331 (4)	0.0443 (14)	
O5	0.0478 (10)	0.1446 (10)	0.6257 (5)	0.064 (2)	
N3	0.1340 (9)	0.1372 (8)	0.5520 (5)	0.0339 (15)	
N1	0.4378 (8)	0.2885 (6)	0.3637 (4)	0.0278 (13)	
N2	0.1021 (8)	0.2377 (6)	0.2836 (4)	0.0282 (12)	
C1	0.6040 (10)	0.3077 (9)	0.4070 (5)	0.0351 (17)	
H1	0.6739	0.2188	0.4357	0.042*	
C16	0.5298 (18)	−0.2934 (13)	−0.0021 (7)	0.064 (3)	
H16	0.4584	−0.3589	−0.0314	0.076*	
C11	0.4407 (11)	−0.0630 (9)	0.1930 (5)	0.0328 (17)	
C14	0.8340 (17)	−0.1765 (12)	0.0162 (8)	0.067 (3)	
H14	0.9697	−0.1615	−0.0009	0.081*	
C10	−0.0635 (12)	0.2054 (10)	0.2451 (5)	0.0349 (18)	
H10	−0.0986	0.1017	0.2507	0.042*	
C8	−0.1339 (12)	0.4738 (10)	0.1900 (6)	0.045 (2)	
H8	−0.2144	0.5531	0.1583	0.054*	
C9	−0.1831 (11)	0.3195 (10)	0.1977 (6)	0.0412 (18)	
H9	−0.2966	0.2933	0.1709	0.049*	
C12	0.5419 (13)	−0.1335 (9)	0.1145 (5)	0.0360 (18)	
C15	0.7274 (19)	−0.2662 (12)	−0.0304 (7)	0.067 (3)	
H15	0.7873	−0.3080	−0.0802	0.080*	
C3	0.5708 (13)	0.5819 (10)	0.3691 (7)	0.050 (2)	
H3	0.6165	0.6812	0.3708	0.060*	
C2	0.6744 (12)	0.4517 (10)	0.4106 (6)	0.0420 (19)	
H2	0.7907	0.4609	0.4406	0.050*	
C13	0.7444 (14)	−0.1082 (10)	0.0878 (6)	0.049 (2)	
H13	0.8178	−0.0458	0.1181	0.058*	
C17	0.4357 (15)	−0.2243 (11)	0.0694 (6)	0.049 (2)	
H17	0.3002	−0.2400	0.0864	0.058*	
C6	0.1514 (10)	0.3891 (7)	0.2771 (5)	0.0268 (14)	
C7	0.0347 (11)	0.5089 (9)	0.2296 (5)	0.0395 (18)	
H7	0.0708	0.6123	0.2246	0.047*	
C5	0.3344 (10)	0.4157 (7)	0.3236 (5)	0.0275 (14)	
C4	0.3976 (13)	0.5674 (9)	0.3244 (7)	0.047 (2)	
H4	0.3258	0.6557	0.2959	0.057*	

### Atomic displacement parameters (Å^2^)

**Table:**

	*U*^11^	*U*^22^	*U*^33^	*U*^12^	*U*^13^	*U*^23^
Pb1	0.02844 (18)	0.01828 (18)	0.0322 (2)	−0.00253 (11)	−0.00343 (11)	−0.00370 (12)
O3	0.039 (3)	0.049 (4)	0.041 (3)	0.004 (3)	−0.007 (2)	−0.008 (3)
O2	0.030 (3)	0.042 (3)	0.053 (4)	−0.014 (2)	0.003 (2)	−0.010 (3)
O4	0.041 (3)	0.060 (4)	0.060 (4)	0.015 (3)	−0.022 (3)	−0.004 (3)
O1	0.048 (3)	0.047 (3)	0.043 (3)	−0.023 (3)	0.007 (3)	−0.019 (3)
O5	0.050 (4)	0.093 (6)	0.056 (4)	−0.025 (4)	0.012 (3)	−0.025 (4)
N3	0.031 (3)	0.029 (3)	0.043 (4)	−0.006 (3)	−0.002 (3)	−0.007 (3)
N1	0.028 (3)	0.020 (3)	0.036 (3)	−0.003 (2)	−0.005 (2)	−0.004 (3)
N2	0.029 (3)	0.024 (3)	0.032 (3)	−0.004 (2)	−0.003 (2)	−0.004 (3)
C1	0.027 (3)	0.033 (4)	0.046 (5)	−0.005 (3)	−0.010 (3)	−0.004 (4)
C16	0.089 (8)	0.062 (7)	0.043 (6)	0.003 (6)	−0.014 (5)	−0.023 (5)
C11	0.034 (4)	0.032 (4)	0.031 (4)	0.006 (3)	0.002 (3)	−0.006 (3)
C14	0.067 (6)	0.055 (6)	0.073 (8)	0.000 (5)	0.030 (6)	0.001 (6)
C10	0.039 (4)	0.032 (4)	0.035 (4)	−0.002 (3)	−0.007 (3)	−0.010 (3)
C8	0.040 (4)	0.040 (5)	0.051 (5)	0.005 (3)	−0.011 (4)	0.008 (4)
C9	0.033 (4)	0.048 (5)	0.044 (5)	−0.005 (3)	−0.011 (3)	−0.006 (4)
C12	0.050 (5)	0.026 (4)	0.031 (4)	0.004 (3)	−0.002 (3)	−0.002 (3)
C15	0.102 (8)	0.057 (6)	0.038 (6)	0.012 (6)	0.012 (5)	−0.022 (5)
C3	0.050 (5)	0.028 (4)	0.076 (7)	−0.010 (4)	−0.017 (4)	−0.013 (4)
C2	0.040 (4)	0.041 (5)	0.051 (5)	−0.012 (4)	−0.008 (4)	−0.019 (4)
C13	0.051 (5)	0.040 (5)	0.056 (6)	−0.008 (4)	0.009 (4)	−0.018 (4)
C17	0.056 (5)	0.050 (5)	0.042 (5)	−0.004 (4)	−0.009 (4)	−0.012 (4)
C6	0.027 (3)	0.021 (3)	0.032 (4)	0.003 (3)	−0.003 (3)	−0.003 (3)
C7	0.042 (4)	0.027 (4)	0.048 (5)	0.000 (3)	−0.004 (4)	0.004 (4)
C5	0.031 (3)	0.022 (3)	0.029 (4)	−0.003 (3)	−0.001 (3)	−0.006 (3)
C4	0.050 (5)	0.023 (4)	0.070 (6)	−0.002 (3)	−0.013 (4)	−0.007 (4)

### Geometric parameters (Å, °)

**Table:**

Pb1—O1	2.432 (5)	C16—C17	1.387 (13)
Pb1—N2	2.441 (6)	C11—C12	1.498 (11)
Pb1—N1	2.471 (5)	C13—H13	0.9300
Pb1—O2	2.619 (6)	C14—H14	0.9300
Pb1—C11	2.863 (7)	C14—C13	1.375 (13)
Pb1—O4	2.871 (6)	C14—C15	1.368 (16)
Pb1—O3	2.928 (6)	C10—C9	1.365 (12)
Pb1—O3^i^	2.893 (6)	C8—H8	0.9300
Pb1—O5^ii^	2.887 (7)	C9—H9	0.9300
O3—N3	1.239 (9)	C10—H10	0.9300
O2—C11	1.252 (9)	C8—C7	1.368 (12)
O4—N3	1.234 (9)	C8—C9	1.378 (11)
O1—C11	1.270 (10)	C12—C17	1.359 (12)
O5—N3	1.251 (9)	C17—H17	0.9300
N1—C5	1.333 (9)	C12—C13	1.397 (12)
N1—C1	1.344 (9)	C3—C4	1.386 (12)
N2—C10	1.334 (10)	C3—C2	1.361 (12)
N2—C6	1.355 (8)	C6—C7	1.386 (10)
C1—C2	1.362 (10)	C7—H7	0.9300
C1—H1	0.9300	C6—C5	1.483 (9)
C2—H2	0.9300	C5—C4	1.399 (10)
C16—C15	1.376 (16)	C3—H3	0.9300
C16—H16	0.9300	C4—H4	0.9300
C15—H15	0.9300		
			
O1—Pb1—N2	85.6 (2)	O2—C11—C12	118.6 (7)
O1—Pb1—N1	80.21 (18)	O1—C11—C12	118.6 (7)
N2—Pb1—N1	66.18 (18)	O2—C11—Pb1	66.1 (4)
O1—Pb1—O2	51.83 (16)	O1—C11—Pb1	57.6 (4)
N2—Pb1—O2	77.48 (18)	C12—C11—Pb1	167.3 (6)
N1—Pb1—O2	121.47 (18)	C15—C14—C13	121.3 (10)
O1—Pb1—C11	26.2 (2)	N2—C10—C9	122.2 (7)
N2—Pb1—C11	83.2 (2)	C7—C8—C9	119.2 (8)
N1—Pb1—C11	102.9 (2)	C10—C9—C8	119.3 (7)
O2—Pb1—C11	25.91 (19)	C17—C12—C13	119.8 (8)
O1—Pb1—O4	152.8 (2)	C17—C12—C11	119.5 (8)
N2—Pb1—O4	72.48 (18)	C13—C12—C11	120.7 (8)
N1—Pb1—O4	76.30 (19)	C14—C15—C16	118.7 (9)
O2—Pb1—O4	133.87 (16)	C2—C3—C4	120.5 (7)
C11—Pb1—O4	153.8 (2)	C3—C2—C1	118.5 (7)
O1—Pb1—O3	139.88 (17)	C14—C13—C12	119.3 (9)
N2—Pb1—O3	110.35 (18)	C12—C17—C16	119.9 (9)
N1—Pb1—O3	73.97 (19)	N2—C6—C7	120.8 (6)
O2—Pb1—O3	164.48 (18)	N2—C6—C5	115.7 (6)
C11—Pb1—O3	162.4 (2)	C7—C6—C5	123.5 (6)
O4—Pb1—O3	43.33 (16)	C8—C7—C6	119.4 (7)
O1—Pb1—O5^ii^	120.12 (20)	N1—C5—C4	121.4 (7)
N2—Pb1—O5^ii^	85.50 (19)	N1—C5—C6	117.2 (5)
N1—Pb1—O5^ii^	144.52 (19)	C4—C5—C6	121.4 (7)
O2—Pb1—O5^ii^	68.42 (19)	C3—C4—C5	117.8 (8)
C11—Pb1—O5^ii^	93.96 (21)	N1—C1—H1	118.6
O4—Pb1—O5^ii^	74.99 (19)	C2—C1—H1	118.6
O3—Pb1—O5^ii^	98.23 (19)	C1—C2—H2	120.8
O1—Pb1—O3^i^	85.60 (19)	C3—C2—H2	120.7
N2—Pb1—O3^i^	149.75 (17)	C2—C3—H3	120.0
N1—Pb1—O3^i^	83.81 (17)	C4—C3—H3	120.0
O2—Pb1—O3^i^	118.16 (17)	H4—C4—C3	121.1
C11—Pb1—O3^i^	100.76 (19)	H4—C4—C5	121.1
O4—Pb1—O3^i^	105.12 (17)	C6—C7—H7	120.3
O3—Pb1—O3^i^	61.80 (17)	H7—C7—C8	120.3
O5^ii^—Pb1—O3^i^	123.69 (18)	H8—C8—C7	120.4
N3—O3—Pb1	95.6 (5)	H8—C8—C9	120.4
C11—O2—Pb1	88.0 (5)	C8—C9—H9	120.4
N3—O4—Pb1	98.6 (4)	H9—C9—C10	120.3
C11—O1—Pb1	96.2 (4)	C9—C10—H10	118.9
O4—N3—O3	120.0 (7)	H10—C10—N2	118.9
O4—N3—O5	120.7 (7)	C12—C13—H13	120.4
O3—N3—O5	119.3 (7)	H13—C13—C14	120.3
C5—N1—C1	119.0 (6)	H14—C14—C13	119.4
C5—N1—Pb1	119.9 (4)	H14—C14—C15	119.3
C1—N1—Pb1	121.0 (5)	H15—C15—C14	120.7
C10—N2—C6	119.1 (6)	H15—C15—C16	120.6
C10—N2—Pb1	119.9 (5)	C15—C16—H16	119.5
C6—N2—Pb1	121.0 (4)	C17—C16—H16	119.6
N1—C1—C2	122.8 (7)	H17—C17—C12	120.1
C15—C16—C17	120.9 (10)	H17—C17—C16	120.0
O2—C11—O1	122.8 (7)		

Symmetry codes: (i) −*x*+1, −*y*, −*z*+1; (ii) −*x*, −*y*, −*z*+1.
